# Exploration in the Mechanism of Action of Licorice by Network Pharmacology

**DOI:** 10.3390/molecules24162959

**Published:** 2019-08-15

**Authors:** Meimei Chen, Jingru Zhu, Jie Kang, Xinmei Lai, Yuxing Gao, Huijuan Gan, Fafu Yang

**Affiliations:** 1 College of Traditional Chinese Medicine, Fujian University of Traditional Chinese Medicine, Fuzhou 350122, China; 2 College of Chemistry and Materials Science, Fujian Normal University, Fuzhou 350007, China; 3 College of Chemistry and Chemical Engineering, Xiamen University, Xiamen 361005, China

**Keywords:** licorice, food additive, network analysis, target identification, KEGG pathway analysis

## Abstract

Licorice is a popular sweetener and a thirst quencher in many food products particularly in Europe and the Middle East and also one of the oldest and most frequently used herbs in traditional Chinese medicine. As a wide application of food additive, it is necessary to clarify bioactive chemical ingredients and the mechanism of action of licorice. In this study, a network pharmacology approach that integrated drug-likeness evaluation, structural similarity analysis, target identification, network analysis, and KEGG pathway analysis was established to elucidate the potential molecular mechanism of licorice. First, we collected and evaluated structural information of 282 compounds in licorice and found 181 compounds that met oral drug rules. Then, structural similarity analysis with known ligands of targets in the ChEMBL database (similarity threshold = 0.8) was applied to the initial target identification, which found 63 compounds in licorice had 86 multi-targets. Further, molecular docking was performed to study their binding modes and interactions, which screened out 49 targets. Finally, 17 enriched KEGG pathways (*p* < 0.01) of licorice were obtained, exhibiting a variety of biological activities. Overall, this study provided a feasible and accurate approach to explore the safe and effective application of licorice as a food additive and herb medicine.

## 1. Introduction

Licorice is a popular sweetener and a thirst quencher found in many soft drinks, food products, and snacks, particularly in Europe and the Middle East. The habit of consumption of such a natural beverage is more popular in hot environments. The traditional concept that licorice is a healthy natural substance without side effects promotes its liberal consumption, which can occasionally be dangerous [1]. Licorice is honored as the reconciler in most Chinese herbal prescriptions, which is one of the oldest and most frequently prescribed herbs in traditional Chinese medicine [2]. Nowadays, licorice extracts, active compounds, and preparations have been used for the treatment of peptic ulcers, liver diseases, Addisonʼs disease, and many other diseases [3,4,5]. Many studies have reported that some active chemical ingredients in licorice possessed antitumor, antimicrobial, antiviral, anti-inflammatory, immunoregulatory, and several other activities that contributed to the recovery and protection of the nervous, alimentary, respiratory, endocrine, and cardiovascular systems [6,7]. For example, the main constituent of licorice, glycyrrhizic acid, of which the sweetness is 200 to 250 times that of sucrose, widely used in various foods, and the sweetness retains for a long time [8]. As a medicine, glycyrrhizic acid holds with bioactivity including neuroprotection, anti-inflammatory, and anti-allergic reactions [9,10]; while the long-term excessive intake of glycyrrhizic acid could cause side effects, such as sodium retention and hypertension [11]. Another major flavone ingredient liquiritigenin, not only can act as an Akt protein kinase inhibitor and a highly potent estrogen receptor agonist [12], but also produce IFN-γ and IL-2 via the CD4+ Th1 immune response, remarkably decreased the production of S. aureus α-hemolysin in S. aureus-mediated lung cell injury and exhibits a significant blood glucose-lowering effect [13,14]. Thus, it is necessary to systematically analyze its potential molecular mechanism and bioactive ingredients of licorice as a food additive due to many chemical ingredients contained in licorice.

Considering the fact that licorice contains hundreds of chemical compounds and acts on multiple cellular targets, it is difficult to systematically study the mechanism of licorice using conventional methods [15]. Therefore, the identification of a complex molecular mechanism is a major challenge in herbal research. Thus, new methods and strategies, such as network pharmacology are urgently needed to address this problem. Network pharmacology, proposed by Andrew L. Hopkins, clarifies the synergistic effects and the underlying mechanism of multi-component and multi-target agents by analyzing various networks of complex and multilevel interactions [16,17]. This method has been widely applied in the systematic understanding of the mechanism of herbal formula [18,19]. For example, Zhu et al. analyzed the molecular targets of clinical Chinese herb medicine for the treatment of metastatic rectal cancer by network pharmacology, which found that 18 herbs out of 295 Chinese herbs were significantly correlated with survival results (*p* < 0.05). They realized the anti-CRC activity mainly through suppressing the biological activities of human epidermal growth factor receptor 2, PPARγ, and Retinoid X Receptor proliferation and vascular endothelial growth factor receptor expression [18]. Gao et al. ascertained molecular targets of herbs prolonging the survival time of patients with advanced hepatocellular carcinoma (HCC) based on this method [19].

In this study, a network pharmacology approach that integrated drug-like evaluation, structural similarity analysis, target identification, KEGG (Kyoto Encyclopedia of Genes and Genomes) pathway analysis and network analysis was established to elucidate the potential molecular mechanism and bioactive ingredients of licorice. First, we collected the structural information of compounds for licorice from the Beilstein/Gmelin CrossFire Chemical database, Chinese Herbal Drug Database and the Handbook of the Constituents in Chinese Herb Original Plants [20,21]. Second, the drug-like property of compounds in licorice was calculated to select compounds with good oral drug-likeness, which were subjected to the initial target prediction based on structural similarity measures with known target ligands in ChEMBL database performed by Open Babel 2.3.1. Third, molecular docking was further employed to study the binding interactions between potential ligands in licorice and targets. Finally, the network analysis and a KEGG enriched pathway analysis was followed to determine the pharmacologic activities of licorice. This study provided a systematical approach for exploration in the potential molecular mechanism of licorice, which was hoped to give some insights into the safe and effective application of licorice as a food additive and herb medicine.

## 2. Results and Discussion

### 2.1. Drug-Likeness Analysis of Licorice

The oral drug-like property of compounds in licorice was assessed by the Lipinski rule of five, that is, molecular weight lower than 500 Da, number of H-bond donors (a_don) less than 5, number of H-bond acceptors (a_acc) less than 10, number of rotatable bonds (b_rotN) lower than 10 and octanol-water partition coefficient (logP(o/w) lower than 5, is of importance for the screening of drugs with pharmacological activity [22,23]. Among 282 compounds in licorice, 181 compounds coincided with the five rules of oral medications. The statistic results of their drug-like property descriptors were listed in Table 1.

### 2.2. Potential Targets and Bioactive Compounds of Licorice

After performing structural similarity measurements of known ligands of targets, compounds with a similarity greater than 0.8 were considered to be bioactive ligands for these targets [24]. Thus, it led to the discovery of 63 compounds from licorice to have 86 putative targets. Further, molecular docking was employed to study binding interactions, which found 63 compounds well interacted with 49 targets with higher dock scores than the cut-off value of −8. Table 2 listed the detailed target information of docking results. Of note, the potential targets of licorice can be roughly divided into 4 categories including enzyme, transcription factor, membrane receptor, and transporter. The biological activity of targets spreads widely, which involves an inflammatory response, metabolism of neuroactive and vasoactive amines, transcription regulation of hormones, glyceraldehyde oxidoreductase activity, putrescine transmembrane transporter activity, neuronal apoptosis, estrogen biosynthesis, retinol metabolism, one-carbon metabolism, oxidation stress, steroid metabolism, lipid metabolism, apoptosis, cell cycle and differentiation, glucocorticoid biosynthesis, insulin signaling cascade, inactivation of the neurotransmitter acetylcholine, purine degradation, melanin biosynthesis, decreased drug accumulation, and so on. Among them, the targets in the neuro-endocrine-immune system accounted most, which acted as a pivot in the regulation of host homeostasis and the natural optimization of health through complex communications between chemical messengers, including hormones, cytokines, and neurotransmitters.

**Figure 1:**
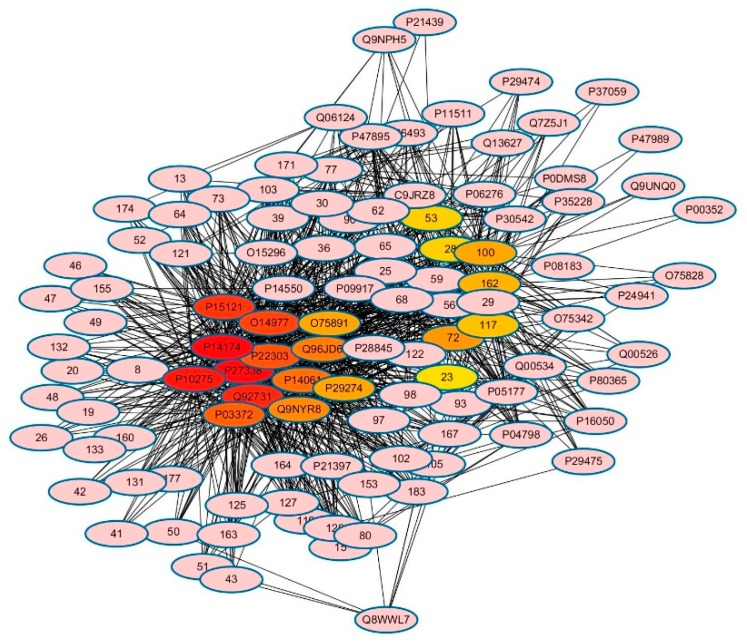
The compound-target network: the Arabic numeral round nodes referred to 63 compounds from licorice; the other nodes represent 49 targets. Color depth for ranking of top 20 hub nodes. The sequence of colors is red-orange-yellow from high ranking to low ranking.

Further to illuminate the relationships between bioactive compounds and hit targets, the compound-target (C-T) network was constructed. As shown in Figure 1, the compound-target network was composed of 112 nodes (63 compounds and 49 potential targets). The network centralization and network heterogeneity were 0.410 and 0.772, respectively, demonstrating that some nodes were more important than others in the network. Table 2 also listed a few simple parameters of the C-T network, such as degree (defined as the number of edges adjacent to the node) and betweenness (defined as the ratio of the number of shortest paths passing through the node among all shortest paths), which have been proposed as metrics in assessing major nodes. Generally, the greater a node’s degree or betweenness is, the more significant the node is in the interactive network [26]. As seen in Table 2, macrophage migration inhibitory factor (MIF) had the largest degree (63) and betweenness (0.0743), indicating that MIF was a crucial target of licorice. MIF, a small pro-inflammatory cytokine, known as a central regulator of innate immunity and inflammation, has been implicated in numerous diseases, such as oncogenesis, arthritis, atherosclerosis, tissue damages and septic shock [27,28]. Our results showed that 63 compounds in licorice can inhibit the activity of MIF, indicating the immunosuppressive effect and anti-inflammation activity of licorice. It provided a clue for the clinical application of licorice in the treatment of respiratory inflammation and immune systems including a host of skin inflammation and allergies [29]. In fact, glycyrrhizic acid and licorice flavonoids had been reported to have anti-inflammatory and anti-allergenic effects [10].

As listed in Table 2, potential targets affected by licorice in the efferent nervous system included amine oxidase [flavin-containing] B (MAOB), amine oxidase [flavin-containing] A (MAOA), acetylcholinesterase, cholinesterase, nitric oxide synthase. MAOB was another potential important target of licorice with degree of 63 and betweenness of 0.0743, mainly found in brain of man, which preferentially inactivated the catechin neurotransmitter. MAOB and MAOA are two subtypes of monoamine oxidase (MAO) that oxidizes neurotransmitters and xenobiotic amines by oxidative deamination and has important functions in maintaining normal mental states [30]. The regulation of MAO activity is crucial in neurodegenerative diseases’ treatment. Nowadays, inhibition of MAO-B activity is known as partial treatment of Parkinson’s and Alzheimer’s diseases [31]. Also, acetylcholinesterase was predicted as potential targets of licorice, which was involved in the termination of pulse propagation transmission by rapidly hydrolyzing the neurotransmitter acetylcholine in numerous cholinergic pathways in the central and peripheral nervous systems. Currently, acetylcholinesterase inhibitors such as donepezil, rivastigmine, and galantamine were the main drugs for improving the cognitive function in Alzheimer’s disease [32]. Our results showed that 63 compounds in licorice can inhibit the activity of MAOB and 52 compounds in licorice can inhibit the activity of acetylcholinesterase. This may give some insights into the memory enhancement and neuroprotective effects of licorice in clinical practices.

Androgen receptor, estrogen receptor beta and estrogen receptor are members of the steroid hormone receptor superfamily, a class of receptors that function through their ability to regulate the transcription of specific genes. Androgen receptor mediates androgen signaling, which is associated with numerous human diseases, including prostate benign hyperplasia and prostate cancer [33]. Currently, anti-androgens are clinically successful for androgen-dependent prostate cancer. Our results showed that 61 compounds in licorice can inhibit the activity of androgen receptor. In fact, some constituents of licorice, such as dibenzoylmethane and glycyrrhizin were reported to exhibit anti-neoplastic effects in prostate cancer cell lines by induction of cell cycle arrest and regulation of androgen receptor expression [34]. Also, licorice has phytoestrogen effects and reduces testosterone levels. Licorice root extracts are often consumed as botanical dietary supplements by menopausal women as a natural alternative to pharmaceutical hormone replacement therapy [35]. Licorice root extract and its major isoflavane, glabridin, exhibited different degrees of estrogen receptor agonism in tissues in vitro and in vivo. Our results showed that 50 compounds in licorice including isoflavane and glabridin can activate the activity of estrogen receptor, showing the estrogen-like activity. Anti-androgen and enhancement of estrogen is the current therapeutic target for prostate cancer [36]. Thus, licorice often used for prostate cancer treatment clinically was owing to its estrogen-like and anti-androgen effects [37].

Overall, among 49 potential targets of licorice predicted by our approach, 16 putative targets including androgen receptor (AR), estrogen receptor beta (ESR2), aldose reductase (AKR1B1), acetylcholinesterase (ACHE), estrogen receptor (ESR1), estradiol 17-beta-dehydrogenase 1(HSD17B1), cytochrome P450 1A2 (CYP1A2), cytochrome P450 1A1 (CYP1A1), nitric oxide synthases(endothelial and brain) (NOS1 and NOS3), cholinesterase (BCHE), NADPH oxidase 4 (NOX4), cyclin-dependent kinase 2 (CDK2), hydroxysteroid 11-beta-dehydrogenase 1-like protein (HSD11B1L), ATP-binding cassette sub-family G member 2 (ABCG2), tyrosinase (TYR), and multidrug resistance protein 1 were reported to be affected by ingredients of licorice [38]. In other words, to the best of our knowledge, our study led to the first discovery that: MIF, ALOX5, ALOX15, NOS2, and ADORA3 are involved in the inflammatory response; CDK1, CDK6, DYRK1A, CCNB3, and CDK3 referred to cell cycle, cell division, and apotosis; AKR1B15, HSD11B2, and HSD17B2 are involved in steroid metabolism; MAOB and MAOA are related with neurotransmitters transformation; ALOX12B, ALOX15B, ALOX15, HSD17B2, and ABCB4 are involved in lipid metabolism; PTPN11 and NOX4 referred to glucose homeostasis, which were implicated by licorice.

**Figure 2:**
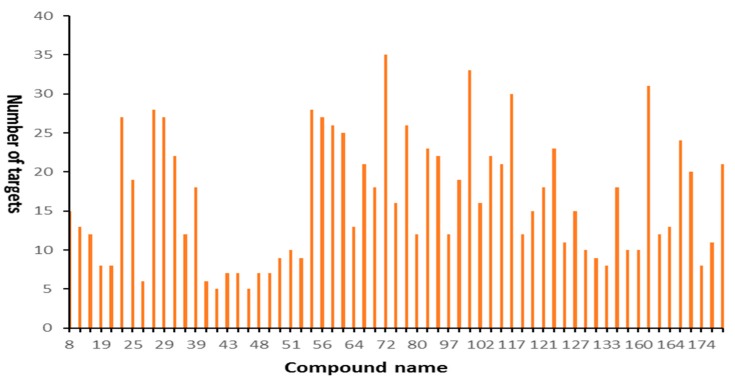
Degree distribution between compounds and putative targets.

The degree of distribution between compounds and putative targets were also presented in Figure 2. On average, each compound had 16 putative targets. Among them, four compounds, such as compounds 72 (lupalbigenin), 100 (sophoraflavone B), 162 (odoratin-7-*O*-beta-d-glucopyranoside), and 117 (ononin) can act on more than 30 targets, and lupalbigenin had the most targets and can simultaneously act on 35 targets followed by sophoraflavone B, odoratin-7-*O*-beta-d-glucopyranoside and ononin. Their chemical names and bioactivity were listed in Table 3. Our study showed that lupalbigenin can simultaneously act on 35 targets, which were widely involved in the biosynthesis and metabolism of neurotransmitters, steroids, anti-inflammatory, anti-tumor, and anti-oxidative stress. Some studies have verified lupalbigenin exhibited anti-tumor activity and anti-dermatophyte activity. They found that lupalbigenin can significantly down-regulate survival proteins, including protein kinase B (pAKT/AKT) and extracellular signal-regulated kinase (pERK/ERK), as well as anti-apoptotic protein B-cell lymphoma 2 (BCL-2) in cancer cells [39]. Ononin was reported to have beneficial biological effects, including anti-inflammatory effects and antioxidant activity [40]. However, the bioactivity of sophoraflavone B and odoratin-7-*O*-beta-d-glucopyranoside has not been reported yet. In general, the compounds with a higher degree of connectivity are more potent pharmacologically. Therefore, these multi-target compounds are of great reference significance for the development of new drugs in the future.

### 2.3. The Potential Pathways Affected by Licorice

**Figure 3:**
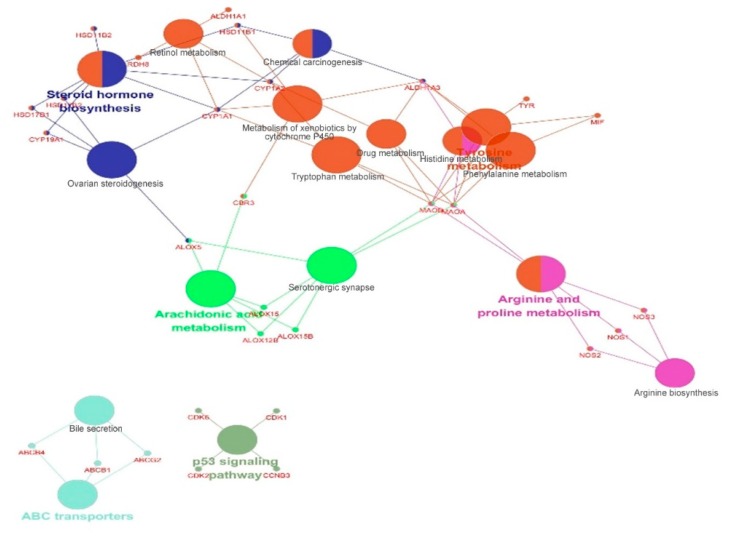
Pathway grouped network for the potential targets of licorice. Pathway grouped network for the potential targets of licorice with terms as nodes linked using ClueGO 2.3.5 analysis, where only the label of the most significant term per group is shown. The node size represents the term enrichment significance and the color of the node reflects the enrichment classification.

Finally, the pathway enrichment analysis with those target proteins was applied to explore potential biological pathways of licorice by using ClueGO 2.3.5 [41]. The results showed that those targets were enriched in 17 pathways with *p* values less than 0.01. We ranked those pathways according to the *p*-value of each enriched pathways in an ascending order (Table 4) for further mechanism analyses. In clinical practices of TCM, licorice has been incorporated into the treatment of various diseases of respiratory system, digestive system, nervous system, and immune system, such as gastric or duodenal ulcers, bronchitis, cough, arthritis, detoxification, viral hepatitis, adrenal insufficiency, depression, psoriasis, allergies, and cancer for thousands of years. Our study indicated that licorice possessed multi-pharmacological effects involved in steroid hormone biosynthesis, tyrosine metabolism, phenylalanine metabolism, ovarian steroidogenesis, arginine and proline metabolism, arachidonic acid metabolism, serotonergic synapse, metabolism of xenobiotics by cytochrome p450, tryptophan metabolism, arginine biosynthesis, histidine metabolism, retinol metabolism, p53 signaling pathway, drug metabolism, chemical carcinogenesis, ABC transporters, bile secretion. The main KEGG pathways can be roughly grouped into 6 categories, shown in Figure 3, including steroid hormone biosynthesis, arachidonic acid metabolism, ABC transporters, p53 signaling pathway, tyrosine metabolism, and arginine and proline metabolism. Among these pathways enriched by those 49 shared targets, we found that the pathway of ranked 1st was closely related with steroid hormone biosynthesis. Steroid biosynthesis is a complex process in which cholesterol is converted to steroid hormones with the involvement of multiple enzymes and cofactors. As listed in Table 4, seven targets were enriched in the pathway of steroid hormone biosynthesis: CYP19A1, CYP1A1, CYP1A2, HSD11B1, HSD11B2, HSD17B1, and HSD17B. Also, ALOX5, CYP19A1, CYP1A1, HSD17B1, and HSD17B2 enriched in the pathway of ovarian steroidogenesis were influenced by licorice. Thus, the anti-oxidation activity, adrenal cortical hormone-like effect and estrogen-like effect of licorice can be partly accounted for.

The normal metabolism of amino acids is an important basis for life activities. As depicted in Table 4, pathways of tyrosine metabolism, phenylalanine metabolism, arginine and proline metabolism, tryptophan metabolism, arginine biosynthesis and histidine metabolism were involved in the pharmacological mechanism of licorice. Four shared targets including ALDH1A3, MAOA, MAOB, MIF were enriched in two pathways of tyrosine metabolism and phenylalanine metabolism, indicating that the main catabolic pathway of phenylalanine is catalyzed to tyrosine by phenylalanine hydroxylase, and then tyrosine can form dopa under the action of tyrosine hydroxylase in the adrenal medulla and nerve tissue, which further becomes a neurotransmitter or hormone after re-decarboxylation, hydroxylation, and methylation. Previous studies demonstrated that decreased dopamine production in brain tissue can lead to Parkinson’s disease [42]. Our results partly supported the clinical application of licorice in Parkinson’s disease. Arachidonic acid is oxygenated and further transformed into a variety of products, which mediate or modulate inflammatory reactions. Generally, aberrant arachidonic acid metabolism is involved in the inflammatory and carcinogenic processes [43]. Our results supported the anti-inflammation activity of licorice targeting ALOX12B, ALOX15, ALOX15B, ALOX5, and CBR3 enriched in the arachidonic acid metabolism pathway for the clinical treatment of inflammation diseases.

The p53 signaling pathway is one of the major apoptosis signaling pathways, which regulates the expression of a wide variety of genes involved in apoptosis, growth arrest or senescence in response to genotoxic or cellular stress. As listed in Table 4, CCNB3, CDK1, CDK2, and CDK6 in the p53 signaling pathway were affected by licorice, exhibited the anti-cancer effect. Also, licorice and its derivatives may protect against carcinogen-induced DNA damage and may be suppressive agents as well [44]. Our results showed that licorice blocked chemical carcinogenesis progress by inhibiting targets of ALDH1A3, CYP1A1, CYP1A2, and HSD11B1. Actually, dibenzoylmethane, a minor phytoconstituent of licorice, has been reported to hold with antioxidant and chemopreventive effects [45]. Besides, our results showed that two pathways of ABC transporters and bile secretion affected by licorice. The ABC transporters are ubiquitous membrane proteins that combines adenosine triphosphate (ATP) hydrolysis with diverse substrates translocations across cell membranes. ABC transporters in the liver are key factors in safeguarding the hepatocyte and avoiding toxicity due to excessive accumulation of bile acid. The biliary secretion of bile acids is critical for multiple liver functions, including digesting fatty nutrients and driving bile flow. When this process is impaired, the accumulated bile acids can cause inflammatory liver damage. Four shared targets including ABCB1, ABCB4, and ABCG2 enriched in these two pathways, indicating that licorice mediated bile acid metabolism by targeting ABCB1, ABCB4, and ABCG2. This result supported the clinical application of licorice in the hepatitis disease. Overall, our pathway analysis results showed that licorice has a variety of biological activities, including anti-oxidation activity, adrenal cortical hormone-like effect and estrogen-like effect, antitumor, anti-inflammatory, neuroprotective effects, and hepatoprotective effects.

## 3. Materials and Methods

### 3.1. Chemical Structures Collection and Oral Drug-Likeness Evaluation

Chemical ingredients from licorice were searched from the Beilstein/Gmelin CrossFire Chemical database, Chinese Herbal Drug Database and the Handbook of the Constituents in Chinese Herb Original Plants [20,21]. In total, we obtained the structural information of 282 compounds for licorice and their 2D structures were sketched using ISIS Draw 2.5 (MDL Information Systems, Inc., San Leandro, CA, USA). Their 3D structures were further minimized using MOE2008 (Chemical Computing Group, Montre) with a Merck molecular force field (Merck Research Laboratories, Boston, MA, USA). The drug-likeness molecular descriptors were calculated to evaluate the drug-likeness property of compounds in licorice based on Lipinski’s rule of five.

### 3.2. Target Prediction and Bioactive Compound Screening for Licorice

First, targets for compounds with good drug-like properties in licorice were predicted based on structural similarity measurements of known target ligands in ChEMBL database using the tanimoto coefficient calculated by Open Bable 2.3.1 [46]. The tanimoto coefficient is the ratio of the atomic pairs shared between two compounds divided by their binding, defined as c/(a + b + c). The variable c is the atomic logarithm of the two compounds, while a and b are the numbers of their unique atom pairs. A good cutoff for biosimilar molecules is 0.7 or 0.8 [47]. Here, compounds in licorice with a similarity greater than 0.8 compared with known ligands of targets were initially considered to be bioactive ligands for these targets.

Second, molecular docking was further applied to filter targets of compounds having similarities greater than 0.8 with ligands of those targets. The crystal structures of 86 initial targets were retrieved from the UniProt databases. After that, the bioactive compounds from licorice were docked to these targets using the MOE2008 as follows. Crystallographic water molecules were removed and hydrogen atoms were added using AMBER99 force field and minimized with a RMSD gradient of 0.05 kcal/mol Å. Then, these target proteins were protonated using AMBER99 force field and minimized with a RMSD gradient of 0.05 kcal/mol Å. Additionally, the binding site was set, based on the ligand atom mode or the automatic atom mode, and the docking placement was searched by the triangle matcher algorithm. Finally, binding interactions between compounds and targets were calculated by the application of two rescoring methods including London dG and Affinity dG, together with a force field.

### 3.3. Network Construction and Analysis

The analysis of the targets, compounds and biological pathways may provide a deep insight into the multi-compounds and multi-pharmacology of licorice. In this study, the screened targets and bioactive compounds together were firstly utilized for compound-target network construction by Cytoscape 3.2.1 [48]. Then, the topological parameters of the network analysis, such as degree and betweenness were calculated to identify key nodes by the network analysis plugin.

### 3.4. KEGG Enrichment Pathway Analysis

The KEGG enrichment analysis of 49 target proteins was conducted to explore the potential biological pathways intervened by licorice using ClueGO 2.3.5 [20], which widely applied in amalgamating the KEGG pathways to construct a functionally efficient pathway network. The localization of the biological and molecular functions of the proteins were detected premised on high confidence with the *p*-value less than 0.01 calculated by the two-side hypergeometric test method. This *p*-value indicates the relative importance of enriched pathways for licorice.

## 4. Conclusions

In this paper, a network pharmacology approach that integrated drug-likeness evaluation, structural similarity analysis, target identification, network construction, and KEGG pathway analysis was successfully established to systematically analyze the potential molecular mechanism and bioactive ingredients of licorice, which can give some insights into the safe and effective application of licorice as a food additive and herb medicine. Our main findings were as follows. First, 181 compounds among 282 compounds in licorice had drug-likeness properties in accordance with the Lipinski rule of five. Second, 86 multi-targets were initially identified based on structural similarity measures with known ligands of targets in the ChEMBL database using the tanimoto coefficient calculation, which also led to the discovery of 63 bioactive compounds in licorice. Third, 49 potential targets were further screened out followed by molecular docking. The bioactivity of 49 targets spreads widely, involving an inflammatory response, neuroactive and vasoactive amine metabolisms, hormone transcription regulation, glyceraldehyde oxidoreductase activity, putrescine transmembrane transporter activity, neuronal apoptosis, oxidation stress, steroid metabolism, lipid metabolism, cell cycle and differentiation, purine degradation, melanin biosynthesis, reduced drug accumulation, and so on. Among them, 16 putative targets were reported to be affected by licorice ingredients. To the best of our knowledge, our study led to the first discovery of inflammatory response regulators such as MIF, ALOX5, ALOX15, NOS2, and ADORA3; cell cycle, division and apotosis factors, such as CDK1, CDK6, DYRK1A, CCNB3, and CDK3; steroid metabolism regulators including AKR1B15, HSD11B2, and HSD17B2; MAOB and MAOA related with neurotransmitters transformation; ALOX12B, ALOX15B, ALOX15, HSD17B2, and ABCB4 involved in lipid metabolism; glucose homeostasis regulators including PTPN11 and NOX4, which were implicated by licorice. Amongst them, the targets in the neuro-endocrine-immune system accounted most, which acted as a pivot in regulating host homeostasis and naturally optimizing health. Four, the network analysis of compounds and targets showed that each compound had 16 putative targets on average among 63 bioactive compounds in licorice. Compounds, such as lupalbigenin, sophoraflavone B, odoratin-7-*O*-beta-d-glucopyranoside, and ononin can act on more than 30 targets, which are of great reference significance for the development of new drugs in the future. Five, the pathway enrichment analysis showed that licorice possessed multi-pharmacological effects involved in steroid hormone biosynthesis, arachidonic acid metabolism, ABC transporters, p53 signaling pathway, tyrosine metabolism, and arginine and proline metabolism.

Overall, our study partly supported the clinical application of licorice in the respiratory system, digestive system, neuro-endocrine-immune system, such as gastric or duodenal ulcers, bronchitis, cough, detoxification, viral hepatitis, adrenal insufficiency, Parkinson’s, psoriasis, allergies, and cancer. Additionally, the results of this study indicated that although licorice is a homologous plant of medicine and food, it contains many biologically active ingredients and is involved in many targets and pathways. In addition to the anti-inflammatory and anti-cancer activities of glycyrrhizic acid (the main ingredient of licorice), many other medicinal chemical ingredients of licorice also have a wide range of activities. Therefore, it is recommended to use licorice at the doctor’s advice and not to take large doses for a long time. Also, the findings in our study will provide rich guidance and reference for the experimental research of licorice in the next stages.

## Figures and Tables

**Table 1:** Drug-like property descriptors of 181 compounds in licorice.

Descriptors	Meaning	Median	Mean	Std. Deviation
Weight	Molecular weight	354.36	354.83	52.93
a_acc	Number of hydrogen bond acceptor atoms	5	4.99	1.41
a_don	Number of hydrogen bond donor atoms	3	2.77	1.14
b_rotN	Number of rotatable bonds	3	3.45	1.81
logP(o/w)	Log of the octanol/water partition coefficient	3.72	3.38	1.34

**Table 2:** Docking results of targets of licorice.

Hit Target [25]	Protein Name	Biological Activity	Between-Ness	Deg-ree	Gene Name
P14174	Macrophage migration inhibitory factor	Immunity, Inflammatory response	0.0743	63	MIF
P27338	Amine oxidase [flavin-containing] B	Neurotransmitters transformation	0.0743	63	MAOB
P10275	Androgen receptor	Transcription regulation of hormone	0.0688	61	AR
Q92731	Estrogen receptor beta	Transcription regulation of hormone	0.0606	59	ESR2
P15121	Aldose reductase	Glyceraldehyde oxidoreductase activity	0.0563	57	AKR1B1
O14977	Antizyme inhibitor protein	Putrescine transmembrane transporter activity	0.0483	53	AZIN1
P22303	Acetylcholinesterase	Neurotransmitters transformation	0.0437	52	ACHE
P03372	Estrogen receptor	Transcription regulation of hormone	0.0400	50	ESR1
P14061	Estradiol 17-beta-dehydrogenase 1	Estrogen biosynthesis	0.0384	49	HSD17B1
Q96JD6	1,5-anhydro-D-fructose reductase	Catalyzes the NADPH-dependent reduction of 1,5-anhydro-d-fructose (AF) to 1,5-anhydro-d-glucitol	0.0271	41	AKR1E2
Q9NYR8	Retinol dehydrogenase 8	Converts all-trans-retinal to all-trans-retinol	0.0211	37	RDH8
P29274	Adenosine receptor A2a	Identical protein binding	0.0170	33	ADORA2A
O75891	Cytosolic 10-formyltetrahydrofolate dehydrogenase	One-carbon metabolism	0.0167	33	ALDH1L1
P47895	Aldehyde dehydrogenase family 1 member A3	Retinol metabolism	0.0101	26	ALDH1A3
P05177	Cytochrome P450 1A2	Oxidation stress	0.0094	25	CYP1A2
C9JRZ8	Aldo-keto reductase family 1 member B15	Estrogen biosynthesis	0.0089	25	AKR1B15
P14550	Alcohol dehydrogenase [NADP(+)]	l-glucuronate reductase activity	0.0088	24	AKR1A1
P04798	Cytochrome P450 1A1	Oxidation stress	0.0048	18	CYP1A1
P28845	Corticosteroid 11-beta-dehydrogenase isozyme 1	Steroid metabolism	0.0044	17	HSD11B1
P09917	Arachidonate 5-lipoxygenase	Inflammatory response	0.0040	17	ALOX5
O75342	Arachidonate 12-lipoxygenase	Lipid metabolism	0.0038	16	ALOX12B
P16050	Arachidonate 15-lipoxygenase	Lipid metabolism, immune and inflammatory responses	0.0038	15	ALOX15
P30542	Adenosine receptor A1	Purine nucleoside binding	0.0032	15	ADORA1
O15296	Arachidonate 15-lipoxygenase B	Lipid metabolism	0.0028	15	ALOX15B
P06493	Cyclin-dependent kinase 1	Cell apoptosis	0.0028	14	CDK1
Q00534	Cyclin-dependent kinase 6	cell cycle and differentiation	0.0025	14	CDK6
P11511	Cytochrome P450 19A1	Oxidation stress	0.0024	13	CYP19A1
Q06124	Tyrosine-protein phosphatase non-receptor type 11	Glucose homeostasis	0.0021	12	PTPN11
P21397	Amine oxidase [flavin-containing] A	Neurotransmitters transformation	0.0015	11	MAOA
P80365	Corticosteroid 11-beta-dehydrogenase isozyme 2	Glucocorticoid biosynthesis	0.0015	11	HSD11B2
Q13627	Dual specificity tyrosine-phosphorylation-regulated kinase 1A	Cell proliferation	0.0013	10	DYRK1A
Q8WWL7	G2/mitotic-specific cyclin-B3	Cell cycle, cell division, meiosis	0.0010	8	CCNB3
Q00526	Cyclin-dependent kinase 3	Cell cycle, cell division, mitosis	0.0008	7	CDK3
P35228	Nitric oxide synthase	Inflammation	0.0006	7	NOS2
Q9NPH5	NADPH oxidase 4	Insulin signaling cascade	0.0006	7	NOX4
Q7Z5J1	Hydroxysteroid 11-beta-dehydrogenase 1-like protein	Oxidation stress, glucocorticoid biosynthesis	0.0005	7	HSD11B1L
P0DMS8	Adenosine receptor A3	Inflammatory response and a suppressive role in osteosarcoma malignancy	0.0005	6	ADORA3
P29474	Nitric oxide synthase, endothelial	Angiogenesis	0.0006	6	NOS3
P29475	Nitric oxide synthase, brain	Neurotransmitter	0.0006	6	NOS1
P24941	Cyclin-dependent kinase 2	Cell cycle, cell division, DNA damage, meiosis, mitosis	0.0004	6	CDK2
P06276	Cholinesterase	Inactivation of the neurotransmitter acetylcholine	0.0003	5	BCHE
O75828	Carbonyl reductase [NADPH] 3	NADPH binding	0.0002	4	CBR3
P37059	Estradiol 17-beta-dehydrogenase 2	Lipid metabolism, steroid biosynthesis	0.0002	3	HSD17B2
Q9UNQ0	ATP-binding cassette sub-family G member 2	Efflux of numerous drugs and xenobiotics	0.0001	3	ABCG2
P00352	Retinal dehydrogenase 1	Retinol metabolism	0.0001	2	ALDH1A1
P21439	Multidrug resistance protein 3	Lipid metabolism	0.0000	2	ABCB4
P47989	Xanthine dehydrogenase/oxidase	Purine degradation	0.0000	2	XDH
P14679	Tyrosinase	Melanin biosynthesis	0.0000	2	TYR
P08183	Multidrug resistance protein 1	Decreased drug accumulation	0.0000	1	ABCB1

**Table 3:** Four compounds with top 4 degree distribution between compounds and putative targets.

Com	Name	Chemical Structure	Bioactivity	Degree
72	lupalbigenin	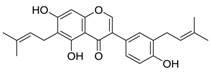	Anti-metastatic activity, anti-dermatophyte activity	35
100	Sophoraflavone B	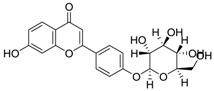	Unreported	33
162	Odoratin-7-*O*-beta-d-glucopyranoside	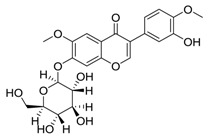	Unreported	31
117	Ononin	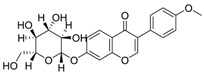	Anti-inflammatory effects, antioxidant Activity	30

**Table 4:** Seventeen KEEG pathways enriched by 49 potential targets of licorice with *p*-value less than 0.01.

ID	Pathway Name	Common Genes	Nr	%Associated Genes	*p*-Value
1	Steroid hormone biosynthesis	CYP19A1, CYP1A1, CYP1A2, HSD11B1, HSD11B2, HSD17B1, HSD17B2	7	12.07	9.17 × 10^−9^
2	Tyrosine metabolism	ALDH1A3, MAOA, MAOB, MIF, TYR	5	14.29	6.05 × 10^−7^
3	Phenylalanine metabolism	ALDH1A3, MAOA, MAOB, MIF	4	23.53	1.08 × 10^−6^
4	Ovarian steroidogenesis	ALOX5, CYP19A1, CYP1A1, HSD17B1, HSD17B2	5	10.00	3.74 × 10^−6^
5	Arginine and proline metabolism	MAOA, MAOB, NOS1, NOS2, NOS3	5	10.00	3.74 × 10^−6^
6	Arachidonic acid metabolism	ALOX12B, ALOX15, ALOX15B, ALOX5, CBR3	5	8.06	1.10 × 10^−5^
7	Serotonergic synapse	ALOX12B, ALOX15, ALOX15B, ALOX5, MAOA, MAOB	6	5.31	1.52 × 10^−5^
8	Metabolism of xenobiotics by cytochrome P450	ALDH1A3, CBR3, CYP1A1, CYP1A2, HSD11B1	5	6.76	2.61 × 10^−5^
9	Tryptophan metabolism	CYP1A1, CYP1A2, MAOA, MAOB	4	10.00	3.82 × 10^−5^
10	Arginine biosynthesis	NOS1, NOS2, NOS3	3	14.29	1.32 × 10^−4^
11	Histidine metabolism	ALDH1A3, MAOA, MAOB	3	12.50	1.99 × 10^−4^
12	Retinol metabolism	ALDH1A1, CYP1A1, CYP1A2, RDH8	4	6.15	2.59 × 10^−4^
13	p53 signaling pathway	CCNB3, CDK1, CDK2, CDK6	4	5.80	3.26×10^−4^
14	Drug metabolism	ALDH1A3, CYP1A2, MAOA, MAOB	4	5.71	3.45 × 10^−4^
15	Chemical carcinogenesis	ALDH1A3, CYP1A1, CYP1A2, HSD11B1	4	4.88	6.31 × 10^−4^
16	ABC transporters	ABCB1, ABCB4, ABCG2	3	6.67	1.30 × 10^−3^
17	Bile secretion	ABCB1, ABCB4, ABCG2	3	4.23	4.80 × 10^−3^
